# Don’t abandon RCTs in IVF. We don’t even understand them

**DOI:** 10.1093/humrep/dez199

**Published:** 2019-11-17

**Authors:** J Wilkinson, D R Brison, J M N Duffy, C M Farquhar, S Lensen, S Mastenbroek, M van Wely, A Vail

**Affiliations:** 1 Centre for Biostatistics, Faculty of Biology, Medicine and Health, Manchester Academic Health Science Centre, University of Manchester, Manchester, UK; 2 Department of Reproductive Medicine, Manchester Academic Health Science Centre, Manchester University NHS Foundation Trust, Manchester, UK; 3 Maternal and Fetal Health Research Centre, Faculty of Life Sciences, Manchester Academic Health Sciences Centre, University of Manchester, Manchester, UK; 4 Nuffield Department of Primary Care Health Sciences, University of Oxford, Oxford, UK; 5 Balliol College, University of Oxford, Oxford, UK; 6 Cochrane Gynecology and Fertility Group, Department of Obstetrics and Gynaecology, University of Auckland, Auckland, New Zealand; 7 Amsterdam UMC, University of Amsterdam, Center for Reproductive Medicine, Amsterdam Reproduction & Development Research Institute, Amsterdam, Netherlands

**Keywords:** infertility, RCT, IVF, statistics, evidence-based medicine, reproductive medicine, machine learning, artificial intelligence, epidemiology, add-on treatments

## Abstract

The conclusion of the Human Fertilisation and Embryology Authority that ‘add-on’ therapies in IVF are not supported by high-quality evidence has prompted new questions regarding the role of the randomized controlled trial (RCT) in evaluating infertility treatments. Critics argue that trials are cumbersome tools that provide irrelevant answers. Instead, they argue that greater emphasis should be placed on large observational databases, which can be analysed using powerful algorithms to determine which treatments work and for whom. Although the validity of these arguments rests upon the sciences of statistics and epidemiology, the discussion to date has largely been conducted without reference to these fields. We aim to remedy this omission, by evaluating the arguments against RCTs in IVF from a primarily methodological perspective. We suggest that, while criticism of the status quo is warranted, a retreat from RCTs is more likely to make things worse for patients and clinicians.

## Introduction

A new narrative in IVF research is emerging

*‘Randomised controlled trials in IVF are dead. They can only tell us whether or not treatments work on average, when what is needed is an assessment of what works in individual patients. They are not suitable for the investigation of complex interventions, such as those comprising assisted reproductive technologies. They are too difficult to perform in subfertile populations, since large numbers of participants are required to show improvements in live birth rates. There are too many confounders in the IVF lab, and the varying skill levels of embryologists make it impossible to determine whether lab-based interventions are effective. As a consequence, the hegemony of the randomised experiment may do more harm than good for people seeking treatment for subfertility, since failure to demonstrate efficacy in an RCT only serves to remove treatment options that may be right for them. Fortunately, it is no longer necessary to rely on the outdated RCT technology in order to evaluate reproductive medical interventions. Advancements in health informatics present the opportunity to amass large amounts of detailed clinical data on the people undergoing IVF, the treatments they receive, and the outcomes of those treatments. These datasets can then be analysed with “powerful algorithms” capable of “exploiting confounders” in order to determine which treatments will work for individual patients. Randomised controlled trials in reproductive medicine are dead. The era of personalised IVF is here’.*

Arguments of this sort have become ubiquitous in medicine and are now being advanced in the field of infertility research (Cohen and Alikani, 2013; Macklon *et al.,* 2019). It is, of course, crucial that we discuss how best to conduct research in IVF, in service of the goal of safely maximizing treatment outcomes. The field of reproductive medicine is a hotbed of innovation. Patients understandably want to give themselves the best possible chance of having a baby, and many clinics trade on this fact to sell add-on interventions with claims of treatment superiority. In this environment, a new treatment can quickly become entrenched if a scientific study appears to support its use. Clearly, it is important that studies evaluating fertility treatments consistently give the right answers. What might be less apparent is the extent to which these discussions hinge upon statistical and epidemiological concepts. In order for these conversations to be fruitful, it is therefore essential that they are conducted with recourse to the sciences of quantitative methodology.

In the following sections, we evaluate the merits of the arguments for observational big data approaches to supplant randomized controlled trials (RCTs) in reproductive medicine from a primarily methodological standpoint. Secondarily, we argue that failure to include methodologists in methodological discussions about the future of comparative effectiveness research is unlikely to produce a robust, relevant evidence base.

## The Troubles with Trials

RCTs in infertility research frequently have methodological weaknesses, limiting their usefulness. We have previously shown, for example, that very few RCTs (and moreover, very few meta-analyses of RCTs) are large enough to detect realistic improvements in live birth rates (Stocking *et al.,* 2019). RCTs included in systematic reviews of Cochrane Gynaecology and Fertility are frequently classified as having a high risk of bias, meaning that there is a distinct possibility that their results are not trustworthy. In fact, a recent review of the evidence behind IVF ‘add-ons’, conducted in the UK by the Human Fertilisation and Embryology Authority (HFEA), concluded that no add-on was supported by high-quality RCT evidence and that none could be recommended (https://www.hfea.gov.uk/treatments/explore-all-treatments/treatment-add-ons/). Critics of RCTs seize on these points to suggest that the scarcity of robust RCTs shows that it is not feasible to do clinical trials in IVF (Macklon *et al.,* 2019). Moreover, they suggest that the failure to demonstrate the effectiveness of new technologies constitutes a proof by contradiction; since trials consistently fail to show benefit of commonly used interventions, it must be that treatment effects in IVF cannot be demonstrated by trials (Cohen and Alikani, 2013; Macklon *et al.,* 2019). (In part this is a misunderstanding of the purpose of the HFEA traffic light system: if add-ons receive sufficient weight of evidence to merit green traffic lights, then eventually many of these will become part of routine treatment. Some treatments having already attained this status may not be considered add-ons at all. Nonetheless, the presence of orange or red traffic lights indicates that these add-ons should not be used in any setting other than a research setting.)

Setting aside the possibility that IVF RCTs are frequently negative because the treatments don’t work, it is often unclear whether critics doubt that RCTs can demonstrate treatment effects in principle or only in practice. We would strongly contest any criticism of randomized experiments *en principe*, but the issue is moot if the weaker claim, that good trials in IVF are not feasible, is correct. The infeasibility of RCTs in IVF may be exaggerated however. The fact that large trials in IVF do, as a matter of fact, take place is one relevant consideration. In fact, in comparison with other specialties, we would suggest that trials in IVF are relatively easy in many respects, particularly for trials requiring only a single treatment cycle. The duration for a single cycle of IVF is short, so that trialists do not have to worry about high levels of non-adherence and attrition over long courses of treatment. Outcome measures are routinely collected as part of care, so it is not necessary to schedule additional visits for additional tests. Despite protestations, the sample sizes required to evaluate treatments are not unusually large (and are perhaps even modest) in comparison with other specialties, and the pool of eligible participants is hardly shallow with 1 500 000 treatment cycles worldwide (European Society for Human Reproduction and Embryology, 2018) and, for example, 68 000 cycles in the UK, annually (Human Fertilisation and Embryology Authority, 2019).

None of this is intended to belittle the considerable efforts of those clinicians, patients and researchers who work very hard to realize randomized studies. Conducting RCTs is difficult and not for the faint-hearted, and so it is not surprising that many studies fall short of the standards required for a definitive treatment evaluation. Rather than motivating the abandonment of RCTs however, we see an imperative to improve them. To this end, there is a substantial and growing body of methodological work aiming to improve the conduct of fertility trials as well as the reporting and analysis of research data (Roberts, 2007; Missmer *et al.,* 2011; Maity *et al.,* 2014; Farland *et al.,* 2016; Griesinger, 2016; Wilkinson *et al.,* 2016; Duffy *et al.,* 2018; Modest *et al.,* 2018; Stocking *et al.,* 2019). Additionally, research on how to make trials easier, for example, by improving methods of recruitment (Treweek *et al.,* 2015; Huang, *et al.,* 2018) and capitalizing on routinely collected data for outcome measurement (Peto *et al.,* 1995; Altman, 2015; Kwakkenbos *et al.,* 2018), is burgeoning. Electronic health records are now used in many clinics, raising the possibilities of automated eligibility signalling and data capture. These innovations might not only improve trials but also make them cheaper, increasing the benefit-cost ratio in both regards. Fewer RCTs in fertility are needed, but collaborative efforts are required to ensure that those that do take place are well-powered, high-quality trials designed to answer the most important questions (Royal College of Obstetricians and Gynaecologists, 2019) with coordinated outcome reporting (Duffy *et al.,* 2018) and methods to facilitate evidence synthesis. An exemplar of this approach is the international consortium of trialists conducting RCTs of fresh versus frozen embryo transfer, using standardized methods in order to allow meaningful pooling of results (e.g. the E-Freeze trial, https://www.npeu.ox.ac.uk/downloads/files/e-freeze/EFeeeze_Protocol_V2.0_18012017.pdf).

## Confounders and Complexity

It has been suggested that RCTs in IVF, particularly for laboratory-based interventions, will fail to detect genuine effects owing to the complex nature of the treatment. Following oocyte retrieval, the oocytes are fertilized, the resulting embryos are cultured and monitored and a decision is made regarding which to transfer. These steps typically involve several embryologists, and the procedures are said to be subject to a large number of ‘confounders’, a phrase used (incorrectly) to describe factors other than the tested intervention which may influence outcomes. Examples include differences in culture media, incubators and air quality (Khoudja *et al.,* 2013; Swain, 2014; Swain *et al.,* 2016). It has also been argued that the varying skill of embryologists both within and across centres introduces ‘technological bias’, since efficacious treatments may not work in the wrong hands (Cohen and Alikani, 2013). These considerations allegedly show that ‘the RCT is not well suited to embryology investigations’ (Cohen and Alikani, 2013).

These discussions relate to sources of variation in RCTs, and so it is regrettable that they have largely been conducted without reference to the long-established science of variation known as ‘statistics’. The design and analysis of experiments to estimate specific effects in the presence of other factors have been the bread and butter of statistical inquiry going back at least as far as Fisher, and the extension of these principles to the study of complex interventions is not new. In particular, study designs and methods of analysis for situations where there is variation in practice or skill between centres and between practitioners (whether these are therapists, surgeons or, indeed, embryologists) are well established (Devereaux *et al.,* 2005; Roberts and Roberts, 2005; Walwyn and Roberts, 2010; Cook *et al.,* 2012; Kahan and Morris, 2013; Roberts and Walwyn, 2013; Sterba, 2017; Senn and Lewis, 2019). Suggestions that these factors preclude valid RCTs in IVF demonstrate a complete lack of awareness of the substantial literature telling researchers exactly how such trials can be done. Thanks to this literature, RCTs accounting for variation between centres and practitioners are not only possible, but take place all the time (Beard *et al.,* 2013; Taylor *et al.,* 2016; Husain *et al.,* 2017; Craig *et al.,* 2018).

In relation to ‘technological bias’, a further suggestion has been that participation in RCTs should be restricted to clinics able to meet certain minimum quality standards (Cohen and Alikani, 2013). Indeed, it is advisable to carry out preliminary, explanatory trials under idealized conditions. However, pragmatic RCTs conducted in real clinical settings are needed to determine whether promising treatment effects survive when transported from a controlled experimental setting to messy reality (Schwartz and Lellouch, 1967). Most do not (Perel *et al.,* 2007; Chalmers *et al.,* 2014; Currie *et al.,* 2019). We have heard objections from device manufacturers that, despite poor showings in pragmatic trials, their intervention works well once clinic staff are given the appropriate training (Munné *et al.,* 2007; Foong *et al.,* 2019). This is a testable claim, with participating clinics allocated in a cluster RCT to receive training in use of the tested intervention. We are not aware of any study demonstrating that the effectiveness of a particular intervention substantively improves with training however. An alternative solution would be to agree to restriction of trial participation to clinics achieving minimum quality standards, provided that device manufacturers similarly agreed not to sell their product to clinics failing to meet these criteria.

## Observational Studies, Powerful Algorithms and Precision

Large, non-randomized studies have been proposed as an alternative to, and perhaps even an improvement over, RCTs in IVF (Macklon *et al.,* 2019). The promise is that large data sizes will allow us to identify not only which treatments are effective but also the subgroups of patients they work for. It is perhaps telling that details of how these studies should be designed and analyzed are generally left to the imagination of the reader. Where scant details are provided, there is little evidence that the requirements for making causal inferences from observational data have been appreciated. Citing the large sample size, an analysis of increasing birth rates over time has been presented as a rebuttal to negative findings in trials of IVF add-ons, requiring a Herculean leap from correlation to causation (Cohen and Alikani, 2013, citing Cohen *et al.,* 2012). Elsewhere, it has been implied that, given datasets of sufficient size and phenotypic detail, the application of ‘powerful’ algorithms will yield the personalized treatment recommendations we seek (Macklon *et al.,* 2019). Unfortunately, the promise of individualized, algorithm-driven IVF is as empty as it is alluring. It may indeed be possible to develop bells-and-whistles algorithms capable of accurately predicting patient outcomes, even though there is no evidence that these methods improve upon good old-fashioned logistic regression at present (Christodoulou *et al.,* 2019). But faced with these hypothetical advances in prediction tools, it is now vitally important that clinicians and researchers equip themselves with a protective mantra: prediction is not causation.

Although we might be able to reliably predict that it is going to rain by observing that many people outside are carrying umbrellas, taking away their umbrellas is not going to stop the rainfall. So, it is for prediction algorithms and observational data; predicting outcomes under a particular treatment tells us nothing about how that outcome would change if we were to treat the patient in a different way, since differences in outcome may in fact be due to differences in patient characteristics or a not-yet-understood underlying biological rationale. Without this information, there is no way to select the best treatment for a given patient, and we should not be surprised if decisions made on this basis are actually deleterious.

The response to this point might be that, with sufficient data, we can use the algorithms to ‘embrace confounding’ (Macklon *et al.,* 2019). This statement makes two important mistakes. First, a great deal of variation in patient outcomes is unexplained and probably forever will be (Rustamov *et al.,* 2017). It is not possible to embrace what we don’t (perhaps can’t) know and can’t measure. Second, there is no algorithm that can take observational data and determine which variables are confounders. In fact, adjusting for some variables that are associated with both treatment and outcome will actually increase bias (Cole *et al.,* 2010; Wilcox *et al.,* 2011). It cannot be stated more plainly: information about which variables to adjust for would not be contained in the data, even if, hypothetically, all confounders were in fact measured.

None of this is to say that well-conducted observational research cannot be extremely valuable and sometimes necessary. It can be unethical to perform RCTs in some scenarios (Braakhekke *et al.,* 2017; Evers, 2017). The literature on study designs and analytic methods for making causal inferences from observational data is highly developed, and there are many thoughtful and compelling applied examples. All of these involve careful pre-data consideration of the causal relationships between the studied variables rather than data-driven algorithms and incorporate both clinical and methodological expertise.

But we might query whether observational studies are appropriate (and to boot, more so than RCTs) for the purposes put forward by critics of trials. Here, the arguments become paradoxical. It is claimed that variation in quality standards and treatment protocols represents an ocean of noise drowning out any treatment signal in RCTs. But in observational databases, a lack of standardization, with respect to treatment, patient selection and measurement protocols, means that the noise is greater than ever. Worse yet, in the absence of randomization, this nuisance variation is associated with treatment allocation, systematically distorting apparent treatment effects. These biases can sometimes be partially offset using causal inference approaches. Unfortunately, small biases invariably remain. This is less problematic for large, stable treatment effects, since the bias would have to be large to fully explain away the result (VanderWeele and Ding, 2017). As such, there is some hope that large effects, such as differences between treatment policies over multiple cycles of IVF, might be imperfectly characterized but nonetheless correctly identified by well-conducted observational studies. On the other hand, these residual biases are devastating for the study of small, variable treatment effects. Of course these are precisely the kinds of effects that we appear to be dealing with in relation to IVF add-ons (Macklon *et al.,* 2019). Large sample sizes, allowing for precise answers, will not save us from bias that leads to inaccurate estimates of effects. There is no value in precise answers if they are precisely wrong. Observational studies are not superior to RCTs for treatment comparisons. This is why the cutting edge in observational research is to mimic an RCT as closely as possible (Sterne *et al.,* 2016; Labrecque and Swanson, 2017).

## Conclusion

There is increasing agreement that the status quo in IVF research is letting patients down, and it is right that all stakeholders should join a conversation about how to change course. However, the conversation so far has largely excluded statistical and epidemiological expertise. The exclusion of methodologists risks steering the ship into the realm of compelling absurdity. Fertility specialists were aghast when a review of add-on therapies offered by fertility clinics mistakenly described surgical sperm selection as an add-on (Heneghan *et al.,* 2016; Spencer *et al.,* 2016). We expect that many methodologists are now similarly dismayed by some of the erroneous claims being advanced by non-experts, both within fertility research and without (Gelman, 2019; Helminen and Reito, 2019).

The abandonment of randomized evidence for algorithmic mining of large datasets will not improve our inferences. Statistics are not that capable, even if we rename them ‘machine learning’. RCTs are challenging, but rather than throw our hands in the air, we believe the answer is to focus efforts on how we can improve them. As we describe above, many of the difficulties that purportedly render RCTs in IVF impossible have already been recognized and resolved. Involvement of the requisite experts could highlight this literature and prevent wasted efforts based on evidential blindspots.

It is crucial that interventions are rigorously evaluated before being offered to patients. The alternative is for treatments of unknown efficacy and safety to be sold using patient demand as a rationale. Shared decision-making is essential, but the onus is on the clinician to bring the facts to the table, and without good quality evidence, patients may feel pressure to roll the dice on add-on therapies rather than be left feeling personally responsible if they don’t become pregnant (Dondorp and de Wert, 2011). In response, critics have suggested that we ‘might be in for a long wait’ if we demand good-quality RCTs before introducing new IVF technologies (Macklon *et al.,* 2019). It is worth reflecting on exactly why that is, since we have suggested that barriers to conducting trials are overblown. A more compelling explanation is the fact that private clinics are able to provide treatments despite a lack of evidence. On the contrary, there can be a commercial drive against robust testing, since a negative trial leaves clinics with one less product to sell.

We have not discussed arguments against RCTs based on their cost. Here, however, the purported advantage of observational studies is also exaggerated. This is particularly true for the kinds of large, prospective databases containing ‘carefully phenotyped cohorts’ created ‘using an expanding array of validated diagnostics’ envisioned by some critics of trials (Macklon *et al.,* 2019). If the idea here is to introduce additional observations or tests that are not routinely collected, then we should not be surprised if this new paradigm is more expensive than conducting robust RCTs. This is a lot to pay to get the wrong answer (Albert, 2013). We suppose here that participants would not be expected to pay for the expanded array of tests themselves, which would open up new conflicts of interest between clinicians and patients.

Regarding the prospects of personalized IVF, the verdict is still out as to whether substantive variation in treatment response exists, and in the absence of empirical evidence, there is a case to be made for focusing on good average medicine in the first instance (Senn, 2016). However, where theory-driven personalized approaches are posited to be effective, RCTs still offer the best modality for evaluating them (Arce *et al.,* 2014; Torrance *et al.,* 2016; Nyboe Andersen *et al.,* 2017) and trial designs for personalized medicine continue to be the subject of methodological research (Senn, 1998; Antoniou *et al.,* 2016*,*^2017^; Araujo *et al.,* 2016). Although it is very much ‘on brand’ for the fertility world to be excited by the advent of new but unproven technologies, the well-conceived and conducted randomized trial, designed to answer an important research question (Royal College of Obstetricians and Gynaecologists, 2019), and reporting core outcome measures to facilitate evidence synthesis (Duffy *et al.,* 2018), is unlikely to be beaten anytime soon. We need better trials and quickly. This will only be realized by close collaboration between experts, including those with lived experience of infertility and IVF. Randomized controlled trials in IVF, as commonly designed and conducted, are dead. Long live the high-quality RCT.

## Authors’ roles

J.W., S.L. and C.M.F. came up with the idea for the manuscript. All authors contributed to writing and editing the manuscript and approved the submitted version.

## Funding

Wellcome Institutional Strategic Support Fund (204796/Z/16/Z) to J.W.

## Conflict of interest

J.W., J.M.N.D., C.M.F., S.L., S.M., M.W. and A.V. are editors for Cochrane Gynaecology and Fertility. J.M.N.D. reports grants from The Royal Society of New Zealand, during the conduct of the study. A.V. has received grants from the Human Fertilisation and Embryology Authority, outside the submitted work. D.R.B. is funded by the NHS as Scientific Director of a clinical IVF service. There are no other conflicts of interest.
